# The role of the hypothalamic-pituitary-adrenal axis in depression across the female reproductive lifecycle: current knowledge and future directions

**DOI:** 10.3389/fendo.2023.1295261

**Published:** 2023-12-12

**Authors:** Liisa Hantsoo, Kathleen M. Jagodnik, Andrew M. Novick, Ritika Baweja, Teresa Lanza di Scalea, Aysegul Ozerdem, Erin C. McGlade, Diana I. Simeonova, Sharon Dekel, Sara L. Kornfield, Michelle Nazareth, Sandra J. Weiss

**Affiliations:** ^1^ Department of Psychiatry & Behavioral Sciences, The Johns Hopkins University School of Medicine, Baltimore, MD, United States; ^2^ Department of Psychiatry, Harvard Medical School and Massachusetts General Hospital, Boston, MA, United States; ^3^ Department of Psychiatry, University of Colorado Anschutz Medical Campus, Aurora, CO, United States; ^4^ Department of Psychiatry and Behavioral Health, Penn State Health, Hershey, PA, United States; ^5^ Department of Obstetrics and Gynecology, Penn State Health, Hershey, PA, United States; ^6^ Department of Psychiatry and Behavioral Sciences, Dell Medical School, University of Texas at Austin, Austin, TX, United States; ^7^ Department of Women’s Health, Dell Medical School, University of Texas at Austin, Austin, TX, United States; ^8^ Department of Psychiatry and Psychology, Mayo Clinic, Rochester, MN, United States; ^9^ Department of Psychiatry, Huntsman Mental Health Institute, University of Utah School of Medicine, Salt Lake, UT, United States; ^10^ Department of Veterans Affairs, Mental Illness Research, Education, and Clinical Center (MIRECC), Salt Lake, UT, United States; ^11^ Department of Psychiatry and Behavioral Sciences, Brain Health Center, Emory University School of Medicine, Atlanta, GA, United States; ^12^ Goizueta Business School, Emory University, Atlanta, GA, United States; ^13^ Center for Women’s Behavioral Wellness, Psychiatry Department, Perelman School of Medicine, University of Pennsylvania, Philadelphia, PA, United States; ^14^ Department of Neuroscience, The Johns Hopkins University, Baltimore, MD, United States; ^15^ Department of Community Health Systems, University of California, San Francisco, San Francisco, CA, United States

**Keywords:** depression, female, hypothalamic-pituitary-adrenal (HPA) axis, perimenopause, peripartum, premenstrual, stress, trauma

## Abstract

The aim of this narrative review is to consolidate knowledge on the role of the hypothalamic-pituitary-adrenal (HPA) axis in depression pathophysiology at different reproductive stages across the female lifespan. Despite growing evidence about the impact of gonadal hormones on mood disorders, no previous review has examined the interaction between such hormonal changes and the HPA axis within the context of depressive disorders in women. We will focus on HPA axis function in depressive disorders at different reproductive stages including the menstrual cycle (e.g., premenstrual dysphoric disorder [PMDD]), perinatally (e.g., postpartum depression), and in perimenopausal depression. Each of these reproductive stages is characterized by vast physiological changes and presents major neuroendocrine reorganization. The HPA axis is one of the main targets of such functional alterations, and with its key role in stress response, it is an etiological factor in vulnerable windows for depression across the female lifespan. We begin with an overview of the HPA axis and a brief summary of techniques for measuring HPA axis parameters. We then describe the hormonal milieu of each of these key reproductive stages, and integrate information about HPA axis function in depression across these reproductive stages, describing similarities and differences. The role of a history of stress and trauma exposure as a contributor to female depression in the context of HPA axis involvement across the reproductive stages is also presented. This review advances the pursuit of understanding common biological mechanisms across depressive disorders among women. Our overarching goal is to identify unmet needs in characterizing stress-related markers of depression in women in the context of hormonal changes across the lifespan, and to support future research in women’s mental health as it pertains to pathophysiology, early diagnosis, and treatment targets.

## 1 Introduction

### 1.1 Background

Women are roughly twice as likely to develop depression compared with men (1, 2), a difference that emerges at puberty (3). Women are at risk for depressive disorders across the lifespan, with unique risk associated with reproductive stages including the menstrual cycle, pregnancy and postpartum, and at perimenopause. Despite the prevalence of depression worldwide, the mechanisms underlying development and severity of depression remain obscure (4, 5). However, one mechanism that has received substantial attention is dysregulation in major stress-related, central homeostatic systems, particularly the hypothalamic-pituitary-adrenal (HPA) axis (4). Among women of reproductive age, ovarian hormones such as estrogen and progesterone interact with the HPA axis (6, 7), making it imperative to study how the dynamic nature of ovarian hormones across the female reproductive lifespan influences HPA axis stress responses and risk for depression (7).

The relationship between reproductive stage and HPA axis function in female depressive disorders can initially be seen in puberty. Onset of major depressive disorder (MDD) is predicted by cortisol hyporeactivity in early stages of puberty versus cortisol hyperreactivity in later puberty in high-risk girls (8). Therefore, puberty may mark a time of ovarian hormone-mediated HPA axis sensitization with stage-dependent effects on depression susceptibility (8). As female development proceeds, there is additional evidence for the importance of the HPA axis, as ovarian hormone fluctuations of the menstrual cycle alter HPA axis sensitivity and function (9, 10). Pregnancy includes major physiological changes to the HPA axis, wherein feedforward glucocorticoid signaling in the placenta leads to hyperactivity of the HPA axis (11). Finally, perimenopause, characterized by erratic ovarian hormone fluctuations, is a stage of the female reproductive lifecycle associated with increased depression susceptibility, especially among women who previously experienced a mood disorder (12, 13). The purpose of this review is to present current knowledge on HPA axis function in depressive disorders during key reproductive transitions. We review HPA axis function as it relates to the menstrual cycle and premenstrual dysphoric disorder (PMDD), depression during pregnancy and the postpartum period, as well as depression during the menopausal transition. Given the significant role of stress, adverse experiences, and trauma in influencing both the HPA axis and risk for depressive disorders, we also address relationships of trauma to both depression and HPA axis function among women at each reproductive stage.

#### 1.1.1 The HPA axis and its physiology

The HPA axis is a complex, yet robust, system. It is composed of a cascade of neuroendocrine pathways that maintain physiological homeostasis in the context of stress. Stress can be defined as a challenge to the homeostasis of the system by any physical or psychological stimuli, which are called stressors. The physiological and behavioral changes that occur as a result of exposure to stressors is called stress response (14, 15). Homeostasis is primarily achieved through control of circulating levels of glucocorticoid hormones, with cortisol being the main downstream glucocorticoid in humans. Cortisol has a multitude of physiological functions (16) including regulation of mood (17). While cortisol itself is released by the adrenal gland, effects of cortisol’s negative feedback on the hypothalamus and anterior lobe of the pituitary gland, autoregulation of the corticotrophin-releasing hormone (CRH) synthesis within paraventricular nucleus (PVN), and positive feedback on the amygdaloid CRH system finely tune stress response (18).

**Figure 1:**
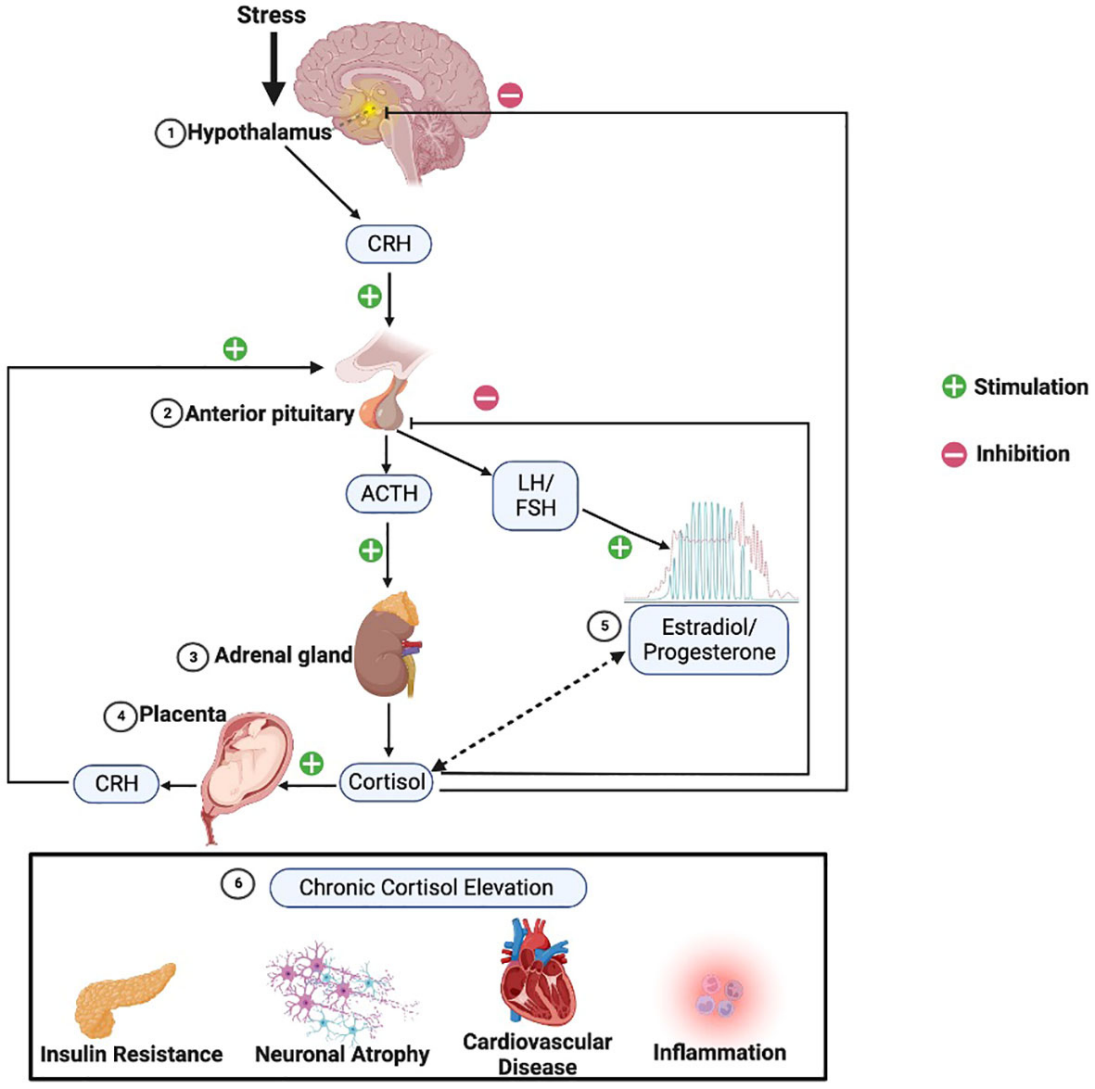
HPA axis physiology. Following stress the 1) hypothalamus triggers release of corticotropin releasing hormone (CRH), which in turn stimulates the 2) anterior pituitary to release adrenocorticotropic hormone (ACTH), which then stimulates the 3) adrenal glands to release cortisol. Cortisol provides negative feedback at the level of the pituitary and hypothalamus to manage excessive cortisol release. However, 4) in pregnant women, the placenta adds an additional positive feedback arm, releasing CRH in response to stimulation from cortisol. 5) Estradiol (red) and progesterone (green) across the female lifespan have varied and incompletely characterized reciprocal interactions with the HPA axis, as indicated by the double-sided dashed arrow. 6) HPA hyperactivity leading to chronically elevated cortisol can result in various metabolic, neuronal, cardiovascular, and immunological changes sometimes associated with depression. (Image created with biorender.com).

Exposure to stress stimulates a series of afferent neural pathways that in turn induce the release of CRH and arginine vasopressin (AVP) from parvocellular neurons of the PVN of the hypothalamus (19). CRH and AVP reach the pituitary gland where they bind to their specific receptors and stimulate the release of adrenocorticotropic hormone (ACTH) from corticotropin cells. ACTH stimulates the adrenal gland to produce and secrete glucocorticoid hormones into general circulation (16). Glucocorticoids operate on their target tissues, including brain, by binding primarily to the glucocorticoid receptor (GR) and the mineralocorticoid receptor (MR), thereby increasing or reducing neuroendocrine secretion throughout the axis (20). As shown in **Figure 1**, glucocorticoids regulate their own production through feedback mechanisms; they inhibit ACTH release at the level of the pituitary gland and also inhibit the release of CRH at the level of the hypothalamus (21, 22). Indirect regulation of HPA activity by glucocorticoids occurs via modulation of the hippocampus, the amygdala, and the prefrontal cortex, which in turn regulate the activity of the PVN (23). Oxytocin (OT) is another neuropeptide that is synthesized in the hypothalamus, secreted from the posterior pituitary gland, and is also involved in regulating stress responses through the HPA axis (24). OT buffers stress as evidenced by reduced salivary cortisol levels, behavioral calmness, and enhanced communication in social stress situations (25, 26) and mediates the mother’s emotional responses toward her child. Its effects can help minimize the effects of stress during and after childbirth (27). CRH concentrations in the peripheral circulation increase most probably due to the CRH coming from the placenta throughout pregnancy. The CRH-binding protein (CRH-BP) that is present in plasma inactivates CRH, and thus may prevent inappropriate adrenal stimulation by the pituitary and keep ACTH within normal limits throughout gestation. CRH-BP levels were shown to fall near term and recover within 48 hours of childbirth allowing the free, potentially bioactive CRH to become available at term to stimulate the release of ACTH from the maternal pituitary (28). The complex interaction between HPA axis and brain serotonergic, noradrenergic, dopaminergic, and gamma-aminobutyric acid(GABA)ergic systems contributes to regulation of the glucocorticoid release. Expression of PVN CRH mRNA is regulated by 5-HT circuits and via 5-HT2C receptors (29). Phasic and tonic GABAergic inhibition, mediated by extrasynaptic δ subunit-containing GABA-A receptors, controls in part the activity of CRH neurons at baseline. Under acute and chronic stress conditions, GABA shows excitatory activity on CRH neurons, and GABAergic inhibition becomes defective. The excitatory effects of GABA following acute stress are specific for CRH neurons in the PVN (30). The interactions among different levels of the HPA axis and the signals generated by the hypothalamic-pituitary-gonadal (HPG) axis, such as estrogens and progesterone, are complex, with an interplay between circulating ovarian and adrenal hormones affecting the intrinsic capability of the system to respond to stressful stimuli (31).

#### 1.1.2 Introduction to the role of stress and trauma in depression via HPA axis mechanisms

As described, exposure to stress stimulates the HPA axis. A history of exposure to chronic stress, adversity, or traumatic experience(s) is a well-established risk factor for developing depression (32–35). The literature on stress, adverse experiences, and trauma sometimes does not provide a clear distinction among these categories of stressors or their associated responses (36–39), and, adopting the continuum perspective (39), we will consider the effects on the HPA axis of this range of negative experiences.

As described above, in healthy responses to acute stress, cortisol functions both as the primary molecule facilitating the stress response and as the main inhibitor of continued HPA axis activity (40, 41). Chronic activation of the HPA axis due to persistent stress can involve sensitized stress responses, chronic basal hypersecretion, or adrenal hyporesponsiveness, and depends upon various factors including the intensity, frequency, modality, and chronicity of the stressor (21). The distinction between traumatic vs. adverse experiences is important. The more-severe category of traumatic experiences has been frequently associated with HPA axis hypoarousal and blunted cortisol reactivity, while the less-severe category of adverse experiences has been associated with HPA hyperarousal and heightened cortisol reactivity (42, 43).

The nature of specific HPA alterations associated with adversity or trauma can be influenced by numerous factors, such as an individual’s genetic profile or specific environmental conditions surrounding the experience (21). One important moderator appears to be the age at which the adverse experience occurred, with childhood experiences having particular salience. Adverse childhood experiences (ACEs) encompass a range of stressors, including chronic stressors such as household dysfunction ((e.g., parental divorce, witnessing domestic violence, parental incarceration, mental illness, or substance use (44, 45)), and trauma such as sexual or physical abuse, in childhood and adolescence (46). In the general population, ACEs have been reported to alter HPA axis function in numerous ways, including both excessive and diminished levels of cortisol secretion as well as impaired negative feedback in response to increased cortisol levels (40, 47–50). It has been proposed that many of these changes closely parallel the neuroendocrine characteristics of depression (47). However, research is inconclusive with respect to defining a consistent profile of altered HPA axis activity due to early stress or trauma (51) and its relationship to depression.

In this review, we consider the relationships of a diverse range of stressful, adverse, and traumatic experiences in both childhood and adulthood to HPA alterations and depression among women at key reproductive stages.

#### 1.1.3 Common methods in studying the HPA axis

Varied methods are used in studies of the HPA axis. Common functions, components, and sources of data are summarized in **Table 1**. *Functions* of the axis reflect its specific roles or dynamics in regulating the body’s homeostatic systems, along with the response to stress. *Components* listed in **Table 1** are constituents of the axis that are frequently studied. *Sources of data* represent the biological substance or method used to obtain data about functions and components of the axis.

**Table 1:** Common functions, components and data sources in HPA axis measurement.

Functions Measured	
Basal Activity	
Negative Feedback	
Circadian Patterns	
Reactivity in Response to Acute Stress	
HPA Regulation Associated with Chronic Stress	
Components Measured	
Hypothalamus	
Pituitary Gland	
Adrenal Gland	
Corticotropin-Releasing Hormone	
Adrenal Corticotropic Hormone	
Cortisol	
Glucocorticoid & Mineralocorticoid Receptors	
HPA Circuitry/Pathways	
Sources of Data	
Blood	
Saliva	
Urine	
Hair	
Neuroimaging	

##### 1.1.3.1 Functions

**Figure 2:**
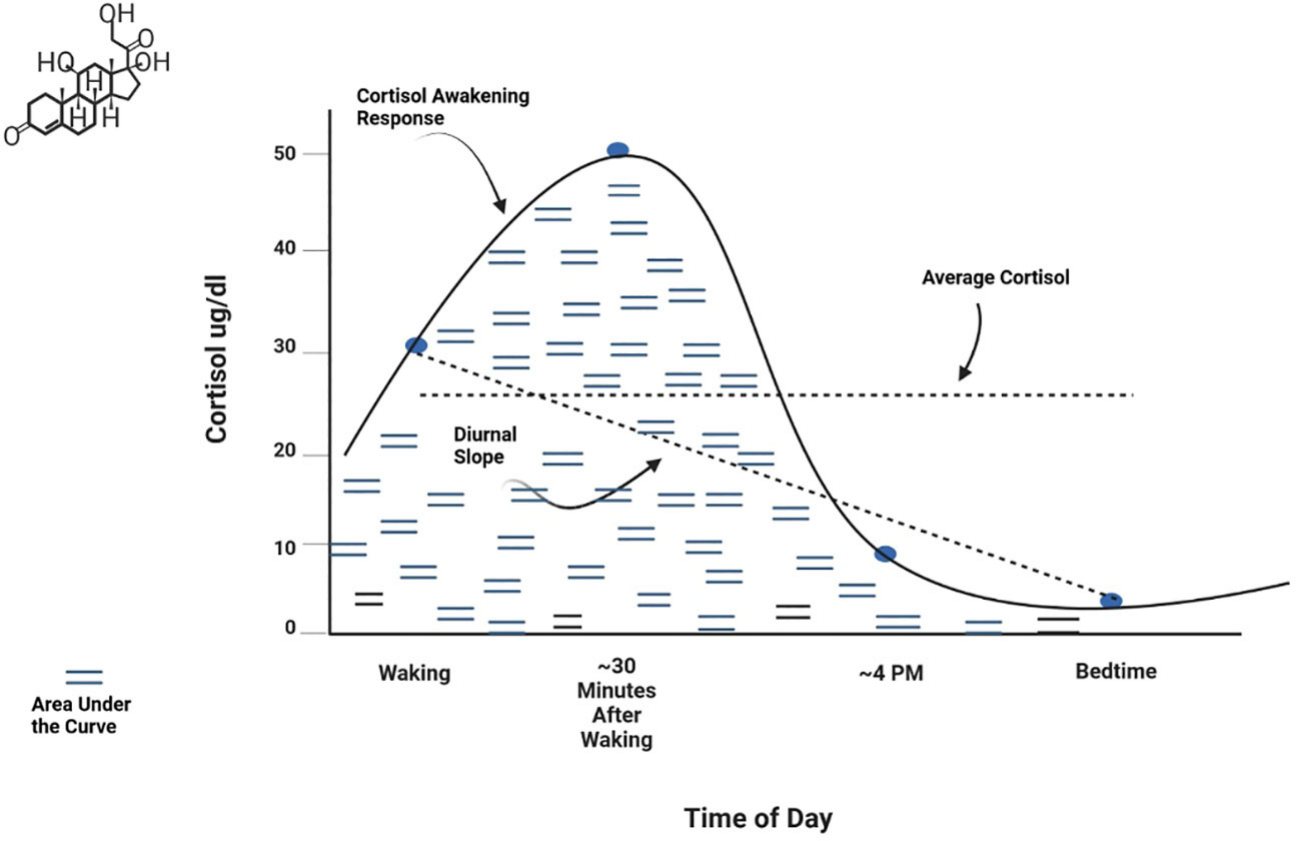
Normative diurnal cortisol pattern.

Basal activity represents the baseline or resting state level of a hormone or other HPA axis component. This is typically measured as an average level at specific time points or across a time period of interest (e.g., 24 hours). Negative feedback refers to the action of cortisol on receptors of the hypothalamus and pituitary gland to regulate production and secretion of hormones in the axis (22, 52). Function of the negative feedback mechanism is frequently measured by assessing cortisol response to pharmacological challenge, that is, administration of corticosteroids such as dexamethasone or prednisolone (agonists of the glucocorticoid receptor) or fludrocortisone (a mineralocorticoid receptor agonist). Circadian patterns are observed levels of hormonal secretion across a 24-hour cycle. Circulating hormones in the axis exhibit strong periodicity throughout the day (53). Basal HPA axis hormones peak in the early morning hours, with the highest peak for cortisol occurring 30-45 minutes after waking. Hormones then decline across the day to a nadir at approximately midnight. Metrics used to measure circadian patterns include the Cortisol Awakening Response (CAR) (the change in cortisol level from waking to about 45 minutes after waking), the Diurnal Slope (the linear change in cortisol levels from waking to sleep), and the overall amount of cortisol secretion across the day [Area Under the Curve (AUC)]. Average cortisol level across the day can also be examined. These metrics are shown graphically in **Figure 2**. Reactivity and regulation in response to stressors are most often measured by examining changes in HPA axis hormones or activity of its circuitry. Reactivity to acute stressors may involve assessment before and after exposure to standardized laboratory stressors (e.g., the Trier Social Stress Test (TSST) and the Montreal Imaging Stress Task) (54, 55) or more naturally occurring stressors for which data is available prior to the occurrence of a stressor and is again assessed post-stressor. Examining HPA axis regulation (or dysregulation) associated with chronic stressors (e.g., domestic violence, caregiving burden, financial hardship, or discrimination) entails assessment of relationships between hormones or other HPA axis components and sustained or persistent stressors over an established time period. It has been suggested that a stressor should persist for at least 6 months to be considered chronic (56), although more stringent definitions may be warranted for specific populations or research aims. Measurement of chronic activation of the HPA axis may involve repeated examination of basal hyper/hyposecretion of hormones or sensitization of hormone or receptor responses, as well as use of specimen sources that inherently measure longer time periods (e.g., hair cortisol) (57). Effective assessment of chronic stress or exposure to cumulative stressors should consider the influence of stressor chronicity, intensity, and frequency on HPA axis function (21).

##### 1.1.3.2 Components

Components of the HPA axis were described previously in Section 1.1 of the Introduction. Their relationships are shown visually in **Figure 1**. The principal glucocorticoid in the axis is cortisol. A large percentage of total cortisol is bound to corticosteroid-binding globulin (CBG), which plays a key role in determining cortisol levels (58). About 3 to 5 percent is free cortisol that is unbound; free cortisol has the capacity to enter cells and affect their function (59). Because of its independence from dynamic alterations in CBG levels and its bioavailability, scientists have proposed that measurement of free cortisol levels may yield a more accurate estimate of the hormone’s status than total cortisol (60, 61). Another factor to consider in assessing cortisol is the regulatory role of receptors: GRs and MRs. When a stress response is initiated, MRs are activated, while GRs play more of a role in regulating recovery from stress (62). The ways in which these different receptors respond (e.g., sensitivity or resistance) and how many MRs versus GRs there may be in tissues will determine the ability to regulate circulating cortisol levels (63, 64). Receptor expression is typically measured by flow cytometry in peripheral leukocytes (65, 66).

##### 1.1.3.3 Sources

Blood has traditionally been a common specimen source for analyzing basal activity of HPA axis hormone levels or expression of MRs and GRs. However, the need for invasive venipuncture in collecting the blood sample makes it less feasible for acquiring data that involve frequent or multiple assessments. Less invasive specimens using dried blood spots are an alternative (67, 68). However, saliva has become increasingly attractive as a sampling source for HPA axis data. Saliva collection is less invasive than blood and yields prolific, easily produced, high-quality samples when collected and assayed correctly (69, 70). Salivary samples are particularly useful for assessment of diurnal circadian patterns that require multiple assessments or pre- and post-responses to acute stressors. Urine is also a non-invasive method to acquire data on basal activity but is more difficult to produce on demand when multiple or repeated measures are needed. Studies continue to provide evidence that salivary and urinary sources provide meaningful alternatives to plasma measurements (71–73).

Over the last decade, hair has become a valuable source for acquiring HPA axis data associated with chronic stress (74, 75) since a 1 cm segment of hair nearest to the scalp reflects the past month’s overall concentration of a hormone (e.g., cortisol) (76). Each additional 1 cm hair segment distal from the first is a measure of the month prior to that. Thus, measures of hormones in hair are thought to represent retrospective HPA axis activation over a designated number of past months.

Lastly, neuroimaging is an essential source of information regarding volume of pituitary and adrenal glands, activation of different components or circuits in the axis, and interactions among various HPA pathways or regions (77, 78). Although volumetric analysis is not common, it is used to determine morphology and normative data [e.g., (79–81)]. Available approaches are magnetic resonance imaging (MRI) and computed tomography (CT) to examine organs and structures, positron emission tomography (PET) scans or functional MRI (fMRI) to assess cellular level activity or functional connections, and magnetic resonance spectroscopy (MRS) for *in vivo* identification and measurement of metabolite concentrations in the brain.

##### 1.1.3.4 Measurement considerations specific to reproductive stages

When studying the HPA axis among women at various reproductive stages, the unique hormonal milieu specific to that stage can influence or confound HPA axis measurement. For instance, the menstrual cycle involves normative changes in glucocorticoid hormone levels and cortisol reactivity at different phases of the cycle, including significantly higher cortisol levels during the follicular phase relative to the luteal phase of the menstrual cycle (9, 10), and greater cortisol reactivity to laboratory-induced stress in the luteal phase (82). Starting in the second trimester of pregnancy, CRH, ACTH, and cortisol begin to rise and then peak prior to delivery of the infant (83). At that time, cortisol levels are 2 to 3 times higher than normal but fall dramatically after delivery (84, 85). Research suggests that HPA axis function continues to change after parturition and likely normalizes by ~3 months postpartum (86), although the possibility of more enduring HPA axis dysregulation is being studied. Lastly, hormonal changes are inherent to perimenopause. The natural transition to menopause exposes women to significant hormonal fluctuations, whereby decreases in progesterone-derived neurosteroids (such as allopregnanolone, a positive modulator of the GABA-A receptor with sedative effects) appear to alter how GABA modulates the HPA axis, ultimately sensitizing perimenopausal women to stress (87). In addition, women transitioning from early to late perimenopause experience increased cortisol as the transition progresses (88, 89). Subsequent sections of this paper provide additional details about normative changes during each reproductive stage.

These natural hormone changes (summarized in **Table 2**) must be considered when studying women’s HPA axis activity and its association with depression. It is essential to control for the specific time point assessed during the reproductive stage and/or build variables related to these hormone fluctuations into measurement models. Such factors may influence the status of HPA axis regulation and be misinterpreted as alterations associated with depressive symptoms, especially among women who are particularly sensitive to hormone change.

**Table 2:** Natural patterns of HPA axis activation during reproductive stages.

Reproductive Stage	Normal HPA Axis Function
Menstrual Cycle	• Greater activation of various axis functions during different phases of the cycle • Strong support for increased cortisol during the follicular phase
Pregnancy	• Incremental increases in axis activation from 12 weeks gestation to birth, especially increased cortisol
Postpartum	• Dramatic decline in axis activation immediately after birth • Continued regulation to normal state by about 3 months
Perimenopause	• Incremental increases in axis activation from early to late menopause, specifically increased cortisol • Glucocorticoid production increases with age

## 2 The role of the HPA axis in depression across the female reproductive lifecycle

### 2.1 The menstrual cycle: introduction and hormonal milieu

**Figure 3:**
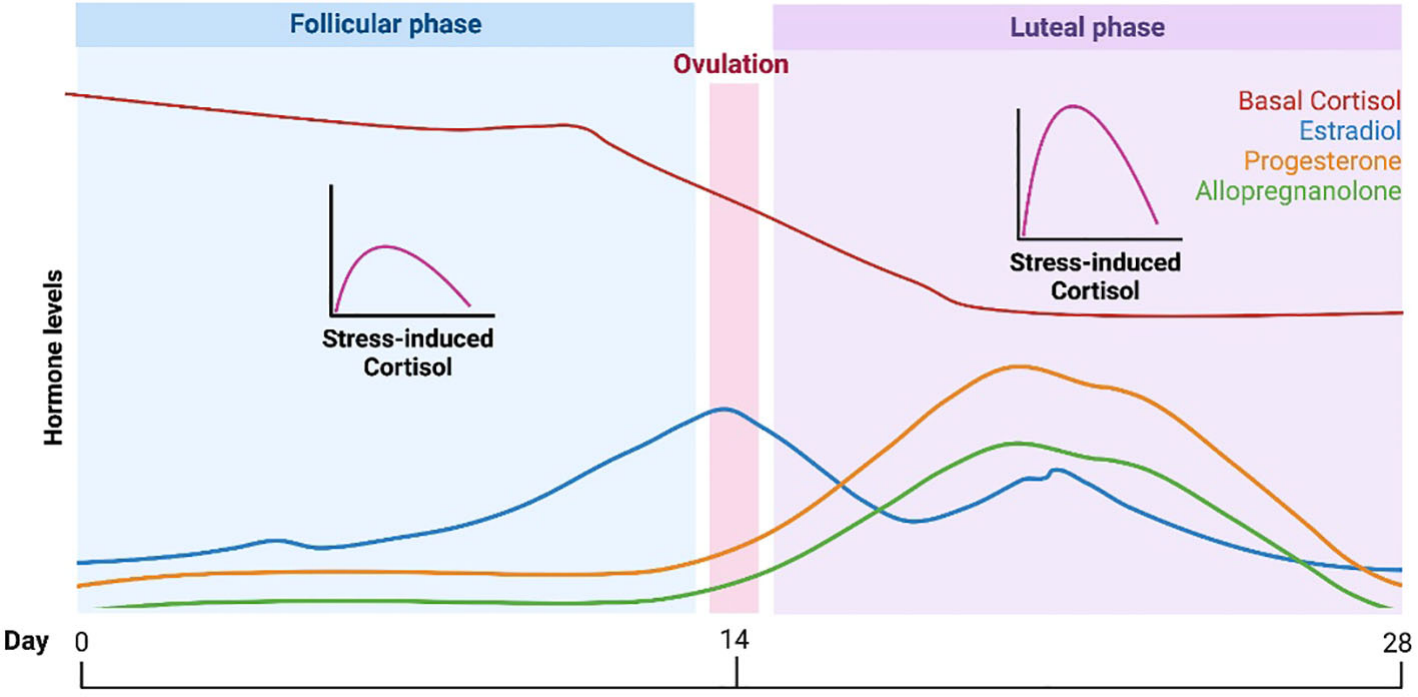
HPA function and the menstrual cycle. Changes in HPA function across the typical 28-day menstrual cycle. During the follicular phase when estradiol and progesterone levels are low, basal cortisol levels are increased while stress-induced cortisol levels are decreased versus the luteal phase. The lower basal cortisol levels in luteal phase is hypothesized to be a function of increased levels of the progesterone metabolite and neuroactive steroid, allopregnanolone.

A vital component of the female reproductive lifecycle is the menstrual cycle. The average menstrual cycle lasts approximately 28 days and is commonly divided into two phases: the follicular phase and the luteal phase (90) (**Figure 3**). During these phases, the major ovarian hormones, estradiol and progesterone, vary according to a predictable pattern (91). Low levels of both estradiol and progesterone characterize the start of the follicular phase, after which estradiol levels begin to rise, reaching a peak around ovulation. This gives way to the luteal phase and rises in progesterone, until both progesterone and estradiol undergo a rapid decline, leading to menstruation and the restart of the cycle.

#### 2.1.1 HPA axis function across the menstrual cycle in healthy females

Studies in animals support definite interactions between the HPG and HPA axes (**Figure 1**), such that changes in progesterone and estradiol across reproductive phases in rodents are associated with differences in HPA activity (6). Past data in humans on the menstrual cycle and the HPA axis have been mixed, but several recent meta-analyses have provided evidence of a consistent pattern. Specifically, higher cortisol levels appear to be present during the follicular phase of the menstrual cycle versus the luteal phase (9, 10). Although a variety of studies and methods of cortisol measurement contributed to the findings in these meta-analyses, the majority of studies used a single measure of total cortisol in blood in the morning (9, 10). The lower cortisol levels in the luteal phase are thought to be due to the relatively higher levels of allopregnanolone, which has inhibitory effects on the HPA axis (92). However, the hypothesized role of allopregnanolone in the luteal phase fails to explain observations that while basal levels of cortisol are lower in the luteal phase, the cortisol response to stress (HPA *reactivity*) is higher (93). One possibility is that decreased tonic inhibition on the HPA axis due to lower cortisol levels in the luteal phase allows for greater increases in cortisol in response to stress.

In addition to the influence of natural variations in hormones across the menstrual cycle, the use of hormonal contraception (specifically, the oral contraceptive pill) results in changes to the HPA axis. Women taking oral contraceptive pills with a combined progestin and estrogen component demonstrate higher levels of basal cortisol (94), with a blunting of cortisol responsiveness to psychosocial stressors (95). This blunting has been attributed to increases in CBG induced by the estrogen component in most oral contraceptive pills, ethinyl estradiol, resulting in a decreased capacity for increases in free cortisol following a stressor (96). Such variations in the HPA axis across the menstrual cycle and due to hormonal contraception argue for consideration of these variables when evaluating HPA-related variables in women.

#### 2.1.2 The HPA axis in premenstrual syndrome (PMS)

Premenstrual syndrome (PMS) involves affective and/or physical symptoms in the luteal phase of the menstrual cycle. While PMS is not a depressive disorder, around 20% of menstruators experience PMS with significant mood symptoms, including depressive features (97, 98). Overall, studies have not provided a clear consensus of HPA axis function in PMS, possibly due to loose and differing definitions of PMS between studies. Compared with controls, women with PMS were found to have lower evening plasma cortisol levels in both menstrual cycle phases (99), higher cortisol levels during the luteal phase (100), attenuated cortisol response to a stressor in both phases (101), attenuated CAR in both phases (102), and lower plasma ACTH levels in the luteal phase (103). However, other studies have found no differences between controls and those with PMS (e.g., in basal salivary cortisol) in either phase (104, 105).

#### 2.1.3 The HPA axis in premenstrual dysphoric disorder (PMDD)

Premenstrual dysphoric disorder (PMDD) is a depressive disorder characterized by mood symptoms that emerge in the premenstrual (luteal) phase of the menstrual cycle and remit in the follicular phase, and is more severe than PMS. PMDD prevalence is estimated at 3-8% of reproductive aged women (106). Studies have found evidence of HPA axis dysregulation in PMDD compared with healthy controls, including lower cortisol levels at baseline and in response to stress in both follicular and luteal phases (107), blunted cortisol response to an exercise stressor in the luteal phase (108), blunted cortisol response to L-tryptophan challenge (109) or serotonin agonist (110) in both phases (110), later cortisol circadian peak in both phases (111), and a delayed CAR peak and a flattened diurnal cortisol slope in both phases (112). Together, these results suggest dampened HPA axis function in PMDD. Further, women with PMDD plus a history of major depression had lower cortisol concentrations than non-PMDD women with a history of major depression, suggesting that PMDD is unique from MDD in terms of physiology (113). Additional evidence of blunted HPA axis function in PMDD is reflected in decoupling of cortisol levels and momentary rumination, while in controls, momentary rumination elicited an increase in cortisol (112). However, other studies have *not* found significant differences between PMDD and controls in HPA axis measures including baseline cortisol (114) and cortisol response to physiological levels of estradiol and progesterone (115). While the above studies suggest dampened HPA axis responsivity in PMDD, a 2016 review reported inconsistent evidence for HPA axis dysregulation in PMDD both at baseline and in response to acute stress (116).

In 2014, Crowley and Girdler proposed that dysregulated ovarian neuroactive steroid control of the HPA axis may be a key mechanism in PMDD (117). Neuroactive steroids are steroid hormones that modulate central nervous system activity, and include ovarian hormones as well as their metabolites. Allopregnanolone is a progesterone derivative and neuroactive steroid that fluctuates across the menstrual cycle in concert with progesterone. Allopregnanolone acts at the GABA-A receptor to produce sedative effects, and modulates the HPA axis in response to stress (118). Crowley and Girdler found that those with PMDD had significantly lower plasma cortisol concentrations at baseline than controls, with concomitantly higher plasma allopregnanolone levels (119). Similarly, there was no difference in diurnal cortisol secretion nor dexamethasone suppression of cortisol between controls and PMDD participants, but those with PMDD plus high serum allopregnanolone levels had blunted nocturnal cortisol levels compared with healthy controls having low allopregnanolone levels (120). Thus, it may be the interaction between the HPG and HPA axes that is involved in PMDD pathophysiology (**Table 3**).

**Table 3:** Summary of HPA axis in the menstrual cycle, PMS, PMDD.

Meta-analyses indicate higher cortisol levels in the follicular phase of the menstrual cycle than the luteal phase.
Hormonal contraceptive pills may blunt HPA axis responsiveness.
In PMS, results on HPA axis measures are mixed, including lower evening cortisol levels, attenuated CAR, and attenuated cortisol response to a stressor in both menstrual cycle phases; higher cortisol and lower ACTH levels in the luteal phase; or no differences in either phase.
In PMDD, studies generally suggest blunted HPA axis function in both phases, including lower cortisol levels at rest and in response to various laboratory stressors, later cortisol circadian peak, delayed CAR peak, and flattened diurnal cortisol slope.

#### 2.1.4 Trauma-related aspects of premenstrual symptoms

##### 2.1.4.1 Role of trauma in premenstrual syndrome (PMS)

The experience of abuse, trauma, or significant stress has been associated frequently with PMS symptoms (121–126). In particular, sexual abuse during childhood or adolescence (122, 127, 128) as well as emotional neglect (129) have shown associations with more severe PMS symptoms. The number and severity of PMS symptoms has been reported to increase in proportion to childhood trauma experiences, with this relationship being mediated by difficulties in emotion regulation (129). However, the role of the HPA axis in this relationship is unclear; quality studies are lacking that compare HPA axis function among females with PMS who have trauma history versus those who do not have trauma history.

##### 2.1.4.2 Role of trauma & HPA axis in premenstrual dysphoric disorder (PMDD)

PMDD has also been associated with a history of abuse or other trauma (124, 129–137). A recent Australian study found that emotional abuse was the most common form of trauma among women with PMDD, reported by 71% (130). As with PMS, the number and severity of PMDD symptoms increase proportionally with childhood trauma experiences, with mediation via emotion regulation dysfunction (129). In a study comparing women with PMDD vs. controls, women with PMDD reporting a history of abuse had lower plasma cortisol than non-abused controls (136), suggesting more suppressed HPA axis activity (135).

### 2.2 The perinatal stage: introduction and hormonal milieu

The perinatal stage is typically defined as the 9 months of pregnancy and approximately 6 months postpartum. Both estrogen and progesterone levels increase during the first trimester of pregnancy and continue to increase, peaking at about 32 weeks gestation (138, 139). At childbirth and transition to the postpartum period, estrogen and progesterone rapidly decline (140). By around 6 months postpartum, these hormones are usually back to pre-pregnancy levels, although breastfeeding and other factors may influence hormonal stabilization (141). Dramatic shifts in gonadal hormone levels across the perinatal period are strongly associated with simultaneous changes in HPA axis function of women (31).

#### 2.2.1 Depression during pregnancy and the postpartum period

Perinatal depression is a term used to describe a major depressive episode that occurs during pregnancy and/or at any time during the year after birth of an infant (postpartum depression). Approximately 15% of pregnant women experience depression (142). Postpartum depression affects about 12% of women who have no prior history of depression (143). A systematic review and meta-analysis found an overall prevalence of 11.9% for depression in women across the entire perinatal period, with prevalence determined via symptom scales being significantly higher than when clinician-based diagnoses were used (144).

#### 2.2.2 Normative alterations of HPA axis function in pregnancy

**Figure 4:**
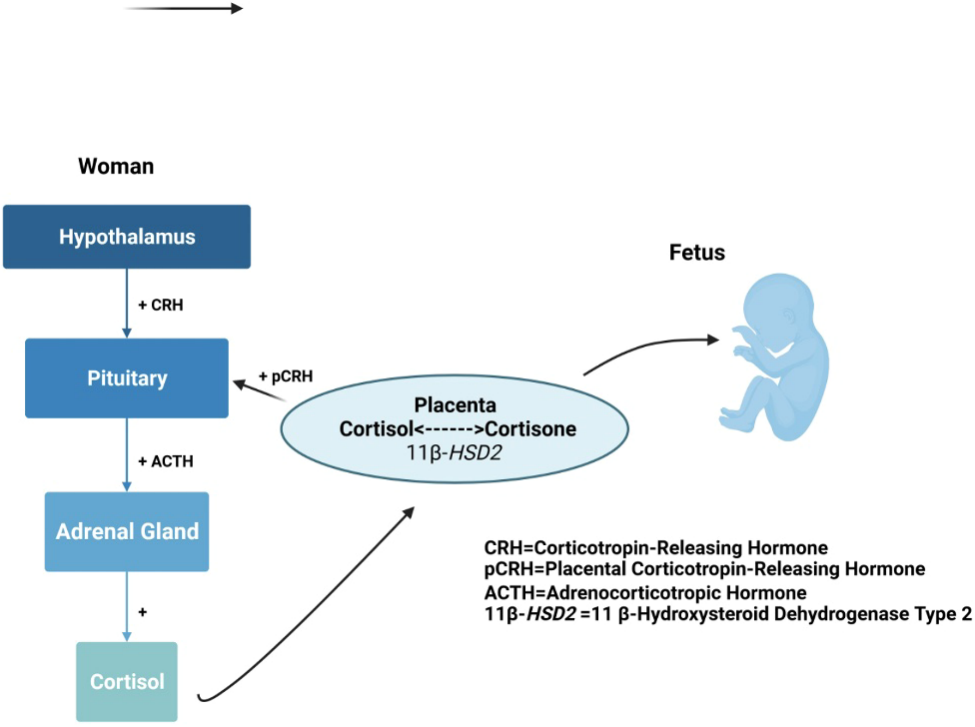
Maternal cortisol stimulates placental release of HPA axis hormones that elicit further maternal cortisol secretion.

The maternal HPA axis undergoes profound changes during the prenatal period (11, 84). The pituitary gland doubles in size, and the production of pituitary peptides increases multifold with progressing gestation. During the third trimester, maternal cortisol levels exhibit great variability and reach approximately 2 to 5 times that of nonpregnant levels (145, 146). During the very last weeks of gestation, a considerable amount of cortisol is produced by the fetal adrenal gland, which probably signals the maturation of fetal organs, as well as the timing of parturition (147). The placenta, however, plays the primary role and contributes to dramatic changes in the stress circuitry (**Figure 1**). The placenta is an endocrine organ of fetal origin. By week 7 of gestation, CRH is synthesized by the human placenta, and is released into the maternal and fetal compartments. Placental CRH (pCRH) production increases dramatically throughout the pregnancy, with high maternal circulation levels mirroring the activity of the hypothalamic-pituitary portal system during stress. In contrast with pregnancy, immunoreactivity of CRH in the plasma is very low or undetectable during a nonpregnant state. Glucocorticoids have inhibitory influence on expression of the CRH gene in the hypothalamus, whereas they activate the promoter region in the placenta and have excitatory influence on the synthesis of CRH. This positive feedback loop results in the dramatic increase in maternal ACTH, cortisol, and pCRH across gestation. As shown in **Figure 4**, pCRH stimulates the woman’s pituitary gland to release ACTH, with downstream effects that further increase a woman’s cortisol level. The difference in functioning of the CRH gene in the hypothalamus and placenta can be explained by expression of different transcription factors, co-activators, and corepressors in these two tissues, thus making the regulation tissue-specific (11, 146).

CBG, which regulates the bioavailability of cortisol in the bloodstream, increases during pregnancy and decreases in the weeks and days before delivery. It is thought that the rise in CBG levels during gestation is insufficient to normalize the pregnancy-induced elevations in free cortisol, possibly due to the high affinity of CBG for increasing progesterone levels (146).

The HPA axis does not operate in isolation. There is a complex interplay among hormones and neurotransmitters during pregnancy. The dramatic increases in cortisol across most of gestation, followed by a decrease after childbirth, significantly impacts other neurological systems, such as the serotonergic, norepinephrinergic, and dopaminergic systems; all notably involved in the pathogenesis of depression (148). Thus, recognition of HPA axis regulation during the transition from pregnancy to postpartum is critical for a better understanding of postpartum depression (149). The acute HPA axis reactivity to stress is dampened in late pregnancy, while the basal levels of CRH, ACTH, and cortisol remain high (145).

#### 2.2.3 HPA axis alterations associated with depression during pregnancy

The CAR, total cortisol output, and the diurnal cortisol slope have each been implicated in depression during pregnancy. Recent systematic reviews indicate that pregnant women who experience depression have higher cortisol levels and a blunted or diminished CAR in contrast with women who have fewer or no depressive symptoms (150–154). Epstein et al. (150) also reported that a greater amount of overall cortisol secretion (AUC) was associated with more depressive symptoms in pregnant women. In addition, two studies (155, 156) found a flatter/dampened cortisol slope (less decline of cortisol from morning to evening) among more depressed pregnant women in contrast with non-depressed. O’Keane et al. (157) reported high sustained evening cortisol output among pregnant women with depression, also resulting in a dampened cortisol decline across the day. In sum, the profile of depression during pregnancy appears to reflect higher levels and overall amount of cortisol secretion, a blunted/dampened cortisol awakening response, and a dampened/flatter cortisol slope (decline) across the day.

#### 2.2.4 HPA axis alterations associated with depression during the postpartum period

The HPA axis profile of depression during postpartum parallels that of pregnancy to a great extent (**Table 4**). During the first 2 months postpartum, most studies report higher cortisol levels associated with depression, including across the entire day (158) as well as in the morning (159) and evening (151, 160). Even women who experience only the ‘postpartum blues’ appear to have higher cortisol levels (84). Higher evening cortisol has been reported at 6 months postpartum as well (161, 162). However, one study during the first month postpartum (163) and another at 12 months postpartum (164) found lower cortisol levels among women who were more depressed. A blunted CAR has also been linked to depression during the first 2 months (165) and at 6 months postpartum (162). Lastly, Scheyer and Urizar (166) reported a flatter diurnal slope at 3 months postpartum. Thus, akin to depression during pregnancy, most research to date indicates higher levels of cortisol, a blunted/dampened CAR, and a flatter diurnal slope among women with postpartum depression (PPD).

**Table 4:** Summary of HPA axis in the perinatal stage (pregnancy and postpartum).

Significant increases in a woman’s own glucocorticoid levels as pregnancy proceeds, combined with increases stimulated by the placenta, result in cortisol levels being 2 to 3 times higher during the 3rd trimester than levels found among nonpregnant women.
Depression during both pregnancy and the postpartum is associated with higher cortisol concentration, a blunted/dampened cortisol awakening response, and a dampened/flatter cortisol slope (decline) across the day than is found for other women.
In pregnancy, women with a history of early-life abuse or trauma show lower mean cortisol levels and reduced cortisol reactivity to stress, compared with women who do not experience trauma; findings for the postpartum are inconsistent.

When considering the trajectory from pregnancy to the postpartum, Scheyer and Urizar (166) found that women who developed PPD had a dampened CAR and a flatter diurnal cortisol slope during pregnancy; the drop in cortisol from the third trimester to 3 months postpartum was also less abrupt for women with depression. There is also evidence for the role of placental hormones in this trajectory, although the data are mixed. Some studies support a relationship between elevated levels of pCRH at midgestation and/or accelerated pCRH trajectories and the development of PPD at 2-3 months postpartum (167–169), while others do not (170).

Alterations in the HPA axis may be due in part to difficulty responding to stressors and maintaining homeostasis in PPD (171). This may be caused by impaired negative feedback that is mediated by cortisol receptors in the anterior pituitary, hypothalamus, and hippocampus, along with ACTH receptors in the anterior pituitary and CRH auto-receptors in the hypothalamus (172). In support of this hypothesis, Gelman and colleagues (172) found that women with PPD exhibit exaggerated responses to the dexamethasone/CRH test as well as a blunted ACTH response to the administration of CRH at 6 - 12 weeks postpartum compared with non-depressed women. A systematic review also reported that studies examining HPA reactivity to stressors suggested an attenuated HPA axis response among women with PPD (173). These findings indicate a hyporeactive HPA axis in regulating acute stress.

#### 2.2.5 Trauma-related aspects of the perinatal stage

##### 2.2.5.1 Pregnancy

To date, several studies have examined the association between ACEs and HPA axis function during pregnancy (as reviewed in Thomas et al., 2018) (174). Shea and colleagues (175) reported that childhood adversity was associated with lower baseline waking cortisol levels, but not the CAR. Bublitz and colleagues also found that childhood sexual abuse was related to lower waking levels of cortisol as well as an increased CAR with both advancing gestation and when early adversity interacted with proximal stressors (176–178). ACEs are also associated with blunted HPA response to stress during pregnancy. Pregnant women with a history of multiple ACEs were less likely to have a cortisol response to a laboratory stressor than women with no or one ACE (179). Pregnant women with a history of ACEs also had lower overall cortisol in response to a mild laboratory stressor compared with pregnant women with no ACE history (180). When assessing lifetime trauma across both childhood and adulthood in an international sample of pregnant women, Dobernecker et al. (181) reported lower hair cortisol concentration, along with increased symptoms of depression, in women with higher trauma load.

In contrast with the significant effects reported for childhood adversity, stress experienced during pregnancy appears to have less impact on HPA axis function. In their scoping review of 48 publications, Rinne et al. (182) found that studies of women affected by stress during the preconception and/or prenatal periods often reported null associations with cortisol or inconsistency in the direction of effects. However, maternal stress experienced during childhood was consistently associated with higher cortisol upon awakening and changes in diurnal cortisol patterns.

In addition to its relationship with HPA axis alterations, a history of ACEs has been associated in multiple studies with increased risk for depression during the prenatal period (183–188). In one large Swedish study, women reporting 5 or more ACEs had a 4.2-fold increased risk of prenatal depression (189). Childhood maltreatment and domestic dysfunction have been identified as two key forms of adversity associated with depression during pregnancy (190). There is also evidence that relational resilience (184) and emotional support (185) may influence the relationship between early adversity and prenatal depression. However, in contrast with the above associations, a multivariate analysis reported no significant correlation of ACE scores and prenatal depression symptoms (191).

##### 2.2.5.2 Postpartum

Few studies have examined effects of prior trauma on HPA axis function during the postpartum. One study found that women with a history of early life trauma had lower mean cortisol levels (192) than other postpartum women. In addition, Brand et al. (193) reported that women with early-life abuse had significantly lower levels of cortisol reactivity in response to a current stressor. This was not supported by another study that reported no differences in cortisol response to a laboratory-based stressor for postpartum women who had a history of ACEs versus women who did not (180). There is emerging evidence that HPA axis changes may be propagated transgenerationally, with the infants of women who were abused having similar neuroendocrine profiles to those of their mothers in the postpartum (193).

### 2.3 Perimenopause: introduction and hormonal milieu

The menopausal transition is the period in a woman’s lifespan involving passage from reproductive potential to ovarian senescence, until permanent cessation of menstruation. The 2011 revised Stages of Reproductive Aging Workshop criteria (STRAW+10) (194) allow standardized classification of the stages leading from reproductive years to postmenopause, passing through the menopausal transition (perimenopause) (194). According to the STRAW+10 staging, the passage from reproductive stage (premenopause) to the early phase of perimenopause (menopausal transition) is marked by the onset of a persistent difference of ≥7 days in the length of consecutive cycles and supportive qualitative hormonal changes (i.e., elevated, but variable, early follicular phase follicle-stimulating hormone (FSH) levels, low anti-Müllerian hormone (AMH), and antral follicular count (AFC)). The late perimenopause is, in turn, defined by amenorrhea of 60 days or longer, and the supportive criteria of circulating FSH levels > 25 IU/L. The end of the 12-month period after the final menstrual period (FMP) has occurred marks the onset of postmenopause, which in its early phase is still characterized by variable FSH and estradiol until approximately 2 years after the FMP, when eventually the levels stabilize in the late postmenopause.

In the absence of sufficient data to adequately define quantitative criteria for hormonal levels or AFC, only qualitative changes are included in the staging system and are referred to as supportive criteria, whereas the onset of each stage is marked by specific menstrual cycle bleeding patterns. Based on data from population-based studies and an international standardized reference values system (195), an exception is represented by the quantitative cut point for FSH levels as supportive criteria for the definition of the late perimenopause (FSH levels > 25 IU) (194).

The progressive loss of ovarian follicular activity and decrease in luteal progesterone and estradiol during menopausal transition result in a weakened negative HPG axis feedback loop, ultimately stimulating increase of the gonadotropins luteinizing hormone (LH) and FSH (194). The hormonal trajectories of the female gonadal hormonal milieu characterizing the years of perimenopause is erratic, with FSH levels highly variable and resulting in similarly highly variable levels of estradiol, until eventually estradiol and progesterone decrease and stabilize approximately two years after the FMP.

#### 2.3.1 Perimenopausal depression

Relative to premenopause, perimenopause is a period of increased depressive symptoms (28%-31% vs. 45%-68% prevalence), as reported by cross-sectional data from six countries (196). Results from three large US longitudinal population-based studies – the Study of Women Across the Nation (SWAN), the Penn Ovarian Aging Study (POAS), and the Seattle Midlife Women’s Health Study (SMWHS) – indicate that women in perimenopause have an estimated increased risk of depressive symptoms ranging from 1.30 to 1.55 in the early transition, and 1.71 to 2.89 in the late transition compared with women in premenopause (196). The SWAN study observed increased risk of MDD recurrence during perimenopause and early postmenopause in women with a previous history of depression (197). Results from SWAN also point to an increased risk of new onset (incidence) syndromal Major Depressive Episode (197).

#### 2.3.2 Normative alterations in HPA axis function during the menopausal transition

Alongside drastic changes in the HPG axis, evidence suggests that the natural menopausal transition is also associated with HPA axis alterations (**Table 5**). Data from the SMWHS (89) describe the prospective changes of first morning urine basal cortisol during perimenopause alongside ovarian hormones, symptoms (i.e., vasomotor, mood, cognitive, sleep symptoms), and stress levels. Investigators reported that *cortisol levels increase during the late perimenopause* (from 7 to 12 months before the late perimenopause to 7 to 12 months after onset of the late perimenopause stage), in line with a rise of FSH levels. However, increased urine cortisol (>10 ng/mg creatinine) during the late perimenopause was *not* associated with mood or other relevant characteristics (e.g., behavioral practices, stressors, vasomotor symptoms, or sleep disturbances).

**Table 5:** Summary of HPA axis in perimenopause.

Evidence suggests that the natural menopausal transition is associated with an increase in cortisol levels during the late perimenopause and that this may not reflect response to stressors but a biological feature intrinsic to the menopausal transition.
The relation between HPA activity related to menopause and to aging itself in women needs to be further elucidated.
Findings of some research suggest that HPA axis dysregulation may underlie the effect of estradiol fluctuation on mood. However, other studies have found no evidence of HPA axis sensitivity to estradiol fluctuation and no HPA changes during the menopausal transition nor in perimenopausal depression.
Perimenopausal depression may not include HPA axis dysregulation as a key pathogenic mechanism, or the pathway by which psychosocial stressors are implicated in perimenopausal depression may not include HPA axis dysfunction.

Subsequent longitudinal data from the same cohort study (198) reported an increase in overnight cortisol from the early to the late perimenopausal transition stage, which was positively associated with measures of stress response (norepinephrine/epinephrine) but not with measures of perceived stress or social stressors. Although, as commented by the authors, dynamic measures of cortisol activity may be more appropriate in detecting relation with stressors, this finding suggested that the rise in overnight cortisol levels may *not* reflect response to stressors, but a biological feature intrinsic to the menopausal transition.

While the above work suggests increases in HPA activity related to menopause, such evidence needs to be considered in the context of the HPA axis hyperactivity related to aging itself, especially in women (199). However, this relation needs to be further elucidated as age during perimenopause may vary and thus perimenopausal and aged women may not be comparable. In a study of participants with age range 20 to 81 years and mean age 49 years, older participants displayed attenuated wake-evening slopes and CAR compared with middle-aged and young adults (200). In another study, the oldest group (65 years and older) had a higher daytime cortisol trajectory compared with the middle-age group (50-64 years), with the latter having a higher trajectory compared with the youngest group (less than 50 years) (201). In a 2005 meta-analysis (202), the cortisol response to challenge was greater in older adults compared with a younger sample, with the effect of age observed to be almost three-fold stronger in women compared with men. Other studies, however, have not observed gender difference in age-related increased cortisol activity (203–205), or reported a greater daytime cortisol trajectory in older men compared with older women (201). Methodological differences among the studies may account for the diverse findings related to the association between gender and cortisol changes.

The increase in HPA activity related to perimenopause has been proposed as associated not only with depression, but also with perimenopausal vasomotor symptoms (206) and poor cognitive performance (207).

Findings on the effects of hormone replacement therapy (HRT) on HPA function are not consistent. In a randomized, placebo-controlled, double-blind trial, three-month treatment of estrogen in healthy postmenopausal women was found to be significantly associated with an elevated serum level of cortisol compared with placebo, whereas in the estrogen plus progesterone replacement therapy group the increase was not significant (208). In a randomized, placebo-controlled, double-blind trial of 6-month combined estrogen and progestin treatment, the 24-hour cortisol serum levels post-treatment was not different in the postmenopausal or premenopausal group compared with placebo.

Transdermal estradiol was found to have a minimal effect compared with oral conjugated equine estrogens (CEE) on the total and free serum concentrations of cortisol and its binding protein (209) as well as inflammatory parameters in a randomized, open-label, crossover study of healthy postmenopausal women (210).

Other hormonal candidates in the relation between menopausal transition and mood changes are androgens; however, the mechanism by which they may interact with estradiol in the pathogenesis of depression remains controversial (211–213).

Low-dose transdermal testosterone as an augmentation strategy was tested in pre- and postmenopausal women ages 21 to 70 with treatment-resistant major depressive disorder in an 8-week randomized, placebo-controlled study (214). Researchers found no significant differences between treatment groups in their effects on depression. Despite being used clinically as an off-label strategy, to date there is consensus that there is not sufficient evidence to support the use of testosterone in women as treatment or preventive strategy of any condition, with the exception of hypoactive sexual desire disorder/dysfunction (215, 216).

#### 2.3.3 HPA axis function in perimenopausal depression

Although individual susceptibility to normal fluctuations of ovarian hormones is considered the major overarching pathophysiological theory behind perimenopausal depression (217–219), the endocrinology of the menopausal transition is not limited to the HPG axis. The HPA axis has been of considerable interest in the physiology of perimenopausal depression based on its well-known contribution to depressive illness vulnerability (220), and given the age-dependent and gender-dependent changes in HPA axis function (88, 199). The mechanism by which HPA axis function may interact with perimenopausal ovarian hormone changes is not fully understood. A recent review (221) — expanding on the heuristic model previously proposed by Gordon et al. (87) — elaborates on the interaction between the HPA and HPG axes, proposing that alongside sensitivity to ovarian hormone changes, women at risk of reproductive mood disorders may also display sensitivity to HPA axis activation. Authors describe the role that estradiol fluctuations, neuroactive steroids, and the GABA-A receptor complex may play in this vulnerability pathway.

Despite these compelling theories, research on the HPA axis in perimenopausal depression is limited relative to other reproductive mood disorders, and the evidence on HPA dysregulation as a major pathogenic factor is controversial (12, 89, 217, 222–224).

Some findings suggest that HPA axis dysregulation may underlie the effect on mood of estradiol fluctuation (223, 225). Gordon et al. (223) examined the relationship between weekly changes in salivary estradiol, salivary cortisol levels, and weekly mood in 30 perimenopausal women with either current depression, past depression, or no history of depression. Greater weekly increases in estradiol were associated with increased cortisol among past and currently depressed women; greater estradiol increases were also associated with negative mood among currently depressed women.

Estradiol may act as an inhibitor of HPA stress response (225); perimenopausal women randomized to 8 weeks of estrogen supplementation experienced reduction in cortisol, ACTH, plasma epinephrine, and norepinephrine responses to stress relative to placebo. This evidence is in line with a recent double-blind, placebo-controlled randomized trial of transdermal estradiol plus intermittent micronized progesterone (TE+IMP) in preventing perimenopausal and early postmenopausal depressive symptoms (226). In contrast, other studies have found *no alterations* in morning cortisol levels associated with perimenopausal depression (217), changes during the menopausal transition but *unrelated* to depressive symptoms (89), or no evidence of HPA axis sensitivity to estradiol fluctuation (12, 224). These results do not support the theory that HPA axis dysregulation plays a role in perimenopausal depression.

A recent study by Gordon and colleagues (224) provided further support of the hypothesis that perimenopausal estradiol fluctuation increases sensitivity to psychosocial stress and vulnerability to depression, although not necessarily via the HPA axis. Among perimenopausal women, weekly fluctuation of estrone-3-glucuronide (E1G), a metabolite of estradiol, was positively associated with anhedonic depressive symptoms, but not associated with cortisol response to a laboratory stressor (the TSST). A similar study of 101 perimenopausal women that assessed weekly estradiol fluctuation via E1G found that mood sensitivity to E1G predicted perimenopausal depression, particularly in women early in the perimenopausal stage without a prior history of depression who reported a low number of baseline stressful life events (SLEs) (12). HPA axis sensitivity to estradiol fluctuation did not predict depressive symptoms or major depression diagnoses during the same time frame.

An experimental study (227) examined HPA axis function in perimenopausal women with and without current or past depression using the combined dexamethasone-corticotropin-releasing hormone (Dex/CRH) test. The two groups did not differ in either baseline or stimulated ACTH, nor cortisol secretion. These results may suggest that – distinct from depressive illness during non-reproductive specific stages – perimenopausal depression may not include HPA axis dysregulation as a key pathogenetic mechanism, or that the pathway by which psychosocial stressors are implicated in perimenopausal depression may not include HPA axis dysfunction.

#### 2.3.4 Trauma-related aspects of the perimenopausal stage

The menopausal transition may be characterized by increased sensitivity to stress, particularly for women with a lifetime history of MDD (196, 228–231). It has been hypothesized that failure to regulate GABAergic tone due to changing levels of neuroactive steroids during perimenopause may cause HPA axis dysfunction, consequently increasing sensitivity to stressors and establishing increased vulnerability to depression (87). Several large longitudinal studies have established associations between past history of stress or trauma exposure and depressive symptoms in the perimenopause. The longitudinal Seattle Midlife Women’s Health Study found that sexual abuse history, and more generally, life stress, were associated with an increased risk of depression during the menopausal transition and early menopause (231). Another longitudinal study following women for nearly 20 years reported that a history of physical abuse or financial instability (experienced in childhood and/or adulthood) was associated with greater depressive symptoms during menopause; however, no such association was found for a history of sexual abuse (232). A number of studies have shown the link between a history of ACEs and depression during menopause. Shea et al. (233) reported that 66% of women seeking treatment for problematic menopause symptoms (including depression) reported a history of ACEs, with higher childhood trauma scores associated with a higher burden of menopausal symptoms. Other studies have shown that the actual number of adverse events during childhood is important to perimenopausal depression risk, with the odds of increased depression severity (234) as well as risk of both lifetime MDD and new-onset MDD at perimenopause (235) being greater as the additional number of ACEs increases. In contrast, a single ACE during childhood has been associated with *reduced risk* of lifetime MDD and menopause-incident MDD (235).

A potential mechanism linking past stress exposure and perimenopausal depressive symptoms is sensitivity to ovarian hormone fluctuations. In a study examining the relationship of lifetime trauma exposure and mood sensitivity to estrogen fluctuations during perimenopause (236), significant interactions between trauma history or SLEs and mood sensitivity to E1G fluctuations were reported. Additionally, a pilot study of women who reported a history of physical or sexual abuse and controls with no history of abuse found an increased risk of depression during perimenopause due to E1G fluctuation (237).

Similar relationships have been established between past stress exposure and depressive symptoms postmenopausally. A longitudinal study of women followed for a median of 19.0 years as part of the SWAN project (197, 238) reported that a history of childhood trauma, including maltreatment, was associated with high postmenopausal depressive symptoms (229). Similarly, interviews from a subsample of 333 women showed that childhood maltreatment, but not current stress, increased the risk of major depression during postmenopause (228).

## 3 Discussion

### 3.1 Key findings and research implications

#### 3.1.1 Key findings for the menstrual cycle

Regarding normative HPA axis function across the menstrual cycle, meta-analyses suggest that basal cortisol levels are higher during the follicular phase than the luteal phase among healthy women (9, 10), potentially due to elevated luteal levels of allopregnanolone inhibiting HPA axis function (92). Conversely, HPA axis reactivity is elevated in the luteal phase, potentially paralleling greater subjective sensitivity to stress in the luteal phase (93). Combined oral contraceptive pills are associated with higher levels of basal cortisol (94) and blunted HPA axis reactivity (95), potentially due to ethinyl estradiol increasing corticosteroid binding protein, producing a decreased capacity for free cortisol increases following a stressor (96).

PMDD is a depressive disorder with symptoms emerging in the luteal phase of the menstrual cycle, and some evidence suggests altered HPA axis function among those with PMDD. Relative to healthy females, individuals with PMDD have altered HPA axis measures in both follicular and luteal phases of the menstrual cycle, including lower cortisol levels at baseline and in response to stress (107, 239), and a delayed CAR peak and a flattened diurnal cortisol slope (112). Notably, some studies have *not* found significant differences between PMDD and controls in HPA axis measures (116). Lack of consistency among studies may be due to failure to consider levels of neuroactive steroids such as allopregnanolone that may influence HPA function (240).

##### 3.1.1.1 Gaps and future directions for menstrual cycle research

Given these variations in HPA axis function across the menstrual cycle, and due to hormonal contraception, researchers should consider menstrual cycle related variables when evaluating HPA axis outcomes in women. Interactions between the HPA and HPG axes, particularly in the reproductive affective disorder PMDD, should be explored in future research. Indeed, deficits in regulation of the HPA axis by GABAergic neuroactive steroids such as allopregnanolone may increase sensitivity to stressors and thus vulnerability to depressive symptoms, particularly among those with PMDD. Importantly, there is little to no work on HPA axis function in premenstrual exacerbation of affective disorders such as major depression. As a large proportion of women with depressive or anxiety disorders experience premenstrual exacerbation of symptoms (241), assessing the role of the HPA axis in this population is a critical gap. Finally, given the potential role of trauma history in PMS and PMDD, more studies are needed that compare HPA axis function among females with premenstrual mood symptoms who have trauma history versus those who do not have trauma history.

#### 3.1.2 Key findings for pregnancy and the postpartum

Profound and dynamic changes occur in glucocorticoid secretion during pregnancy that increase vulnerability of women to depressive symptoms, including in the postpartum. These changes are driven by both maternal hypothalamic and placental hormones. Although all perinatal women experience hormonal alterations, women with depressive disorders appear to have elevated glucocorticoid hormones in both pregnancy and the postpartum. In addition, evidence indicates basal impairments in their glucocorticoid circadian system, as evidenced by a dampened/blunted CAR and a flatter diurnal cortisol slope. Studies also indicate that women with perinatal depression may have an attenuated or hyporeactive HPA axis response to acute stressors.

##### 3.1.2.1 Gaps and future directions for perinatal research

This review suggests a number of needed areas for future perinatal research. First, little is known about how changes in the HPA axis are related to depression over the entire course of pregnancy, including the trajectory of HPA axis alterations from the first to third trimesters. Similar studies are needed over the first year postpartum since emerging evidence indicates that depression may persist through 12 months after delivery. It will be critical to identify factors that may enhance sustained HPA axis alterations associated with depression across the 12 months. Second, investigators need to examine the influence of HPA axis alterations during pregnancy on development of postpartum depression to better understand the entire perinatal trajectory of HPA axis-related depression. Emerging research indicates that a woman’s HPA axis profile during pregnancy may influence her risk of depression in the postpartum. Third, research is scarce regarding the links between and among various components of the HPA axis in the development of perinatal depression. Most research to date has focused on only one component of the axis (e.g., CRH or cortisol) per study, limiting knowledge of where variations from the norm may originate in the axis, how they evolve as they progress through the axis, and their role in development and severity of depression. Fourth, existing evidence implicating HPA axis hormones as potential biomarkers to screen and diagnose perinatal depression is inconsistent, in part due to methodological heterogeneity across studies. Future research should aim to reconcile conflicting findings across studies. In addition, while studies indicate that the use of stress reduction interventions can alleviate maternal stress and reduce morning cortisol levels, future research is needed to study the benefits of interventions that modulate HPA axis activity in the prevention and reduction of depression during pregnancy and the postpartum. Lastly, few studies have examined the bidirectional and interactive influences of placental and maternal hypothalamic glucocorticoid hormones on one another. How these distinct but interdependent neuroendocrine systems may contribute to a woman’s depression risk is not known. This hypothalamic-placental intersection is a unique and essential focus for future perinatal depression research.

#### 3.1.3 Key findings for the perimenopause

Available data do not support the hypothesis that HPA axis dysregulation plays a role in the development of perimenopausal depression. Findings of some research suggest that HPA axis dysregulation may underlie the effect of estradiol fluctuation on mood. However, other studies have found no evidence of HPA axis sensitivity to estradiol fluctuation and no HPA changes in perimenopausal depression, or HPA changes during the menopausal transition but unrelated to depressive symptoms. Moreover, the first study to date reporting on the ACTH activation test in perimenopausal depression found no difference in either baseline or stimulated ACTH and cortisol secretion between women with and without depression. These data may suggest that perimenopausal depression does not include HPA axis dysregulation as a key pathogenetic mechanism, or that the pathway by which psychosocial stressors are implicated in perimenopausal depression may not include HPA dysfunction.

##### 3.1.3.1 Gaps and future directions for perimenopausal research

This review suggests that research on the role of HPA in perimenopause is limited compared with other reproductive stages. Further investigation is warranted to elucidate the role of the HPA axis in the pathogenesis of perimenopausal depression and clarify if the pathway by which psychosocial stressors are implicated includes HPA dysfunction (e.g., comparing HPA markers with other markers of the stress response). Moreover, research is needed to deepen knowledge of the relationships among changes related to menopause itself, perimenopausal depression, and age-related changes in cortisol and the HPA axis, along with implications for health outcomes in women.

Future work with larger samples is also needed to elucidate the mechanisms of depression during the perimenopausal stage, and to understand the influence of trauma history, particularly ACEs, on the development of mental health disorders in later life. Accounting for individual hormonal sensitivity and diverse depression symptom profiles will facilitate personalized treatment strategies (242).

#### 3.1.4 Key findings for trauma-related research

Experiences of stress, adversity, and trauma will affect most women during their lifetime. This review indicates that these experiences, particularly early in life, appear to place women at greater risk for premenstrual affective disorders, perinatal depression, and perimenopausal depression. However, the nature of associations between trauma history and women’s depression is not fully clear, nor are the etiological mechanisms that result in increased risk for depression. Persistent stress that results in chronic activation of the HPA axis can cause sensitized stress responses, chronic basal hypersecretion, or adrenal hyporesponsiveness throughout the female lifespan, and may be associated with an increased risk of depression. In the premenstrual phase, women reporting a history of abuse have lower plasma cortisol than non-abused women, with depression as a potential mediating factor. During pregnancy, women with experiences of early life adversity show lower hair cortisol levels, lower waking levels of cortisol, increasing CAR with advancing gestation, and blunted cortisol response to stress; these HPA axis abnormalities are associated with depression during pregnancy. In contrast, stress experienced during pregnancy is less consistently associated with HPA axis function. Similar to the antepartum period, postpartum women with a history of early-life abuse or trauma show lower mean cortisol levels and reduced cortisol reactivity to stress, compared with women who do not experience trauma. In the perimenopausal stage, ovarian hormone fluctuations have been hypothesized to associate previous exposure to stress with depressive symptoms. Studies have reported an increased risk of depression during perimenopause due to E1G fluctuation, as well as significant interactions between trauma history or stressful life events and mood sensitivity to E1G fluctuations. No HPA axis alterations have been linked to trauma in perimenopausal depression.

##### 3.1.4.1 Gaps in and future directions for trauma-related research

Future research with larger sample sizes should elucidate the factors that interact to influence risk for and development of depressive disorders, and to understand whether the impact of trauma has unique effects in women that result in depression. There are mixed findings regarding the HPA axis in response to stressful, adverse, and traumatic experiences; more careful delineation of underlying mechanisms is needed. The HPA axis profiles of women with a history of traumatic experience in childhood vs. adulthood who subsequently develop depression are presently not well characterized and require further research (51, 243, 244). The differential effects of various types of traumatic experiences (e.g., interpersonal vs. collective trauma) on the development of depression at various reproductive stages should be more thoroughly studied.

### 3.2 Conclusion

Our review indicates that depression experienced by women at different reproductive stages may have vastly different associations with HPA axis regulation (**Table 6**). For instance, depression specific to the menstrual cycle has been associated with lower basal cortisol levels during both the follicular and luteal phases as well as a blunted cortisol response to stressors across both phases. In contrast, depression during both pregnancy and the postpartum is linked to elevated glucocorticoid levels, although (akin to depression specific to the menstrual cycle) women’s response to stress is blunted or attenuated. Contrary to the significant HPA axis relationships with depression found for both the menstrual cycle and perinatal period, there is little evidence for any link between perimenopausal depression and HPA axis dysregulation. Despite these differences in findings across reproductive stages, there is one clear commonality: women undergo dramatic glucocorticoid and ovarian hormonal changes during each stage that must be adjusted for when studying the relationship of women’s HPA axis activity to depression. In addition, our review demonstrates the robust association of early and lifetime trauma to depression at all reproductive stages as well as with lower or suppressed cortisol levels among women experiencing reproductive transitions. However, there is little research to date that has examined the moderating role of trauma in the relationship between the HPA axis and depression among women at any reproductive stage.

**Table 6:** Summary of key points.

This review presented mechanisms of natural HPA function versus HPA alterations associated with depression during each female reproductive stage.
Depression experienced by women at different reproductive stages may have vastly different associations with HPA axis dysregulation. Alterations in the HPA axis may be more strongly associated with premenstrual and perinatal depression than perimenopausal depression.
Women undergo dramatic glucocorticoid and ovarian hormonal changes during different reproductive stages and across the menstrual cycle, that must be adjusted for when studying women’s HPA axis activity and its association with depression.
Experiences of stress, adversity, and trauma will affect most women during their lifetime, and these experiences can alter HPA axis function, placing women at greater risk for premenstrual affective disorders, perinatal depression, and perimenopausal depression.
The age at which adversity is experienced often has a significant effect on HPA axis function, with adverse childhood experiences typically showing the most significant, consistent, and enduring relationships to women’s depression.
Better understanding of the trajectory of changes in the HPA axis over the entire course of pregnancy and postpartum, with a special focus on the unique hypothalamic-placental intersection, is important for future perinatal depression research.
Research on the role of the HPA axis in perimenopause is limited compared with other reproductive stages. The interaction between age-related changes in cortisol/HPA axis and perimenopausal depression with its implications for health outcomes in women is a subject for future research.
Research on the moderating role of trauma in the relationship between the HPA axis and depression among women at any reproductive stage is scarce.
The diversity of study designs to date presents a complex landscape of heterogeneous results that are not conclusive and warrant caution when generalizing.
Future research will need to determine whether unique HPA axis profiles for women at different reproductive stages show validity and reliability, with an ultimate goal of developing assessment tools and treatment of depression targeting women at a specific reproductive stage.

Future research will need to determine whether unique HPA axis profiles for women at different reproductive stages show validity and reliability over time. This may require studying women at these stages with the same standardized designs and protocols. In addition, it will be essential to examine how the nuanced neuroendocrine milieus of each reproductive stage may create a context for the differential HPA axis profiles that have emerged from previous research. Also important is the need to characterize differences in specific depressive symptoms that could potentially play a role in the HPA axis variations observed at each stage (245). Further, impact on HPA axis parameters by treatments for depression at various reproductive stages — for instance, low–dose selective serotonin reuptake inhibitors for PMDD, or brexanolone for postpartum depression — should also be explored in future research. Ultimately, development of such knowledge can inform assessment and treatment that is targeted specifically for women with depressive disorders at a specific reproductive stage rather than assuming that similar approaches are appropriate for all.

In this narrative review, we integrated existing research regarding the HPA axis in the pathophysiology of depression across the reproductive stages of adult women. We also summarized what is known about the relationships among traumatic experience, HPA axis function, and depression during each reproductive stage. Although this review provides an important foundation for further research, the diversity of study designs to date presents a complex landscape of heterogeneous results that are not conclusive and warrant caution when generalizing. Future research will require larger samples, more standardized designs across studies with rigorous controls for confounds, and inclusion of diverse populations of women based on race and ethnicity, degree of economic security or hardship, clinical comorbidities, gender and sexual identity, and other important factors. Understanding the integral relationships between hormones and circuitry of the HPA axis and the HPG axis in women’s depression is a primary need of research at each reproductive stage. Lastly, using large datasets and advanced computational strategies, such as machine learning, may facilitate the identification of risk factors and characteristics for different forms of depression in women, advancing efforts to achieve personalized medicine approaches for the prevention, diagnosis, and treatment of women’s depression. Ultimately, the goal of this research is to develop more effective markers for assessment and targeted treatment that are uniquely appropriate for women at each reproductive stage.

## Author contributions

LH: Conceptualization, Project administration, Supervision, Writing – original draft. KJ: Conceptualization, Project administration, Supervision, Writing – original draft. AN: Conceptualization, Visualization, Writing – original draft. RB: Conceptualization, Writing – original draft. TL: Conceptualization, Writing – original draft. AO: Conceptualization, Visualization, Writing – original draft. EM: Conceptualization, Writing – original draft. DS: Conceptualization, Investigation, Writing – original draft. SD: Conceptualization, Writing – original draft. SK: Conceptualization, Writing – original draft. MN: Writing – original draft. SW: Conceptualization, Investigation, Supervision, Writing – original draft.

## Glossary

**Table:**

ACTH	Adrenocorticotropic hormone/Adrenocorticotropin
AFC	Antral follicular count
AMH	Anti-Müllerian hormone
AUC	Area under the curve
AVP	Arginine vasopressin
CAR	Cortisol awakening response
CBG	Corticosteroid-binding globulin
CEE	Conjugated equine estrogens
CRH	Corticotropin-releasing hormone
CT	Computed tomography
CTQ	Childhood Trauma Questionnaire
Dex/CRH test	Dexamethasone-corticotropin-releasing hormone test
DHEA	Dehydroepiandrosterone
DHEA-S	Dehydroepiandrosterone sulfate
E1G	Estrone-3-glucuronide
E2	Estradiol
FSH	Follicle-stimulating hormone
FMP	Final menstrual period
fMRI	Functional magnetic resonance imaging
GABA	Gamma-aminobutyric acid
GC	Glucocorticoid
GR	Glucocorticoid receptor
HPA	Hypothalamic-pituitary-adrenal
HPG	Hypothalamic-pituitary-gonadal
HRT	Hormonal replacement therapy
IMP	Intermittent micronized progesterone
IU	International units
L	Liter
LH	Luteinizing hormone
pCRH	Placental corticotropin releasing hormone
MDD	Major depressive disorder
MIDUS	Midlife in the United States Study
MR	Mineralocorticoid receptor
MRI	Magnetic resonance imaging
MRS	Magnetic resonance spectroscopy
pCRH	Placental corticotropin-releasing hormone
PET	Positron emission tomography
PMDD	Premenstrual dysphoric disorder
PME	Premenstrual exacerbation
PMS	Premenstrual syndrome
POAS	Penn Ovarian Aging Study
PPD	Postpartum depression
PTSD	Post-traumatic stress disorder
PVN	Paraventricular nucleus
SLE	Stressful life event
SMWHS	Seattle Midlife Women’s Health Study
SWAN	Study of Women Across the Nation
STRAW+10	2011 revised Stages of Reproductive Aging Workshop criteria
TE	Transdermal estradiol
TE+IMP	Transdermal estradiol plus intermittent micronized progesterone
TSH	Thyroid stimulating hormone
TSST	Trier Social Stress Test
